# *Wolbachia*-Virus interactions and arbovirus control through population replacement in mosquitoes

**DOI:** 10.1080/20477724.2022.2117939

**Published:** 2022-10-07

**Authors:** Thomas H Ant, Maria Vittoria Mancini, Cameron J McNamara, Stephanie M Rainey, Steven P Sinkins

**Affiliations:** a Centre for Virus Research, University of Glasgow, Glasgow, UK; b Polo d’Innovazione di Genomica, Genetica e Biologia, Terni, Italy

**Keywords:** Wolbachia, arbovirus, dengue, mosquito, aedes

## Abstract

Following transfer into the primary arbovirus vector *Aedes aegypti*, several strains of the intracellular bacterium *Wolbachia* have been shown to inhibit the transmission of dengue, Zika, and chikungunya viruses, important human pathogens that cause significant morbidity and mortality worldwide. In addition to pathogen inhibition, many *Wolbachia* strains manipulate host reproduction, resulting in an invasive capacity of the bacterium in insect populations. This has led to the deployment of *Wolbachia* as a dengue control tool, and trials have reported significant reductions in transmission in release areas. Here, we discuss the possible mechanisms of *Wolbachia*-virus inhibition and the implications for long-term success of dengue control. We also consider the evidence presented in several reports that *Wolbachia* may cause an enhancement of replication of certain viruses under particular conditions, and conclude that these should not cause any concerns with respect to the application of *Wolbachia* to arbovirus control.

## Introduction

*Wolbachia pipientis* are alphaproteobacteria found in many arthropod species and some nematodes [1]. As obligate endosymbionts, *Wolbachia* are transmitted from mother to offspring in the egg cytoplasm during oogenesis. *Wolbachia-*host interactions often combine elements of parasitism and mutualism: in some instances, they provision a host with nutrients [2], enhance germline stem cell proliferation and thereby increase host fecundity [3], and can have a potent capacity to protect hosts from some pathogens (see Table 1). Many strains also induce one of several forms of reproductive manipulation, facilitating their invasion and maintenance in host populations [47,107–110]. Cytoplasmic incompatibility (CI) is one such manipulation, and results from a sperm modification that causes infertility when a *Wolbachia-*carrying male mates with a *Wolbachia*-free female or with a female carrying a reciprocally incompatible *Wolbachia* strain, but can be rescued by a female carrying the same or otherwise compatible *Wolbachia* strain. Because CI results in a relative reproductive advantage for *Wolbachia-*carrying females, CI-inducing *Wolbachia* strains can often maintain very high population infection frequencies. The fitness advantage for *Wolbachia-*carriers generated by CI is frequency dependent and is highest when a *Wolbachia* strain is close to fixation, while at very low frequencies the population-level effects of CI are negligible. This results in a threshold frequency above which there is spread and below which *Wolbachia* is lost; the threshold depends on CI penetrance, efficiency of maternal transmission, and fitness effects. Phenotypes that improve the spread of a *Wolbachia* strain are expected to be selected for; mathematical models suggest that pathogen protection can significantly lower the frequency thresholds required for *Wolbachia* invasion [111].

**Table 1.** The interactions between various *Wolbachia* strains and virus in different host species. Viral genome type has been color-annotated. The different virus abbeviations are as follows: Dengue virus (DENV); Zika virus (ZIKV); Chikungunya virus (CHIKV); Yellow Fever virus (YFV); West Nile virus (WNV); Mayaro virus (MAYV); Semliki Forest virus (SFV); Cell-fusing agent virus (CFAV); Kunjin virus (KUNV); Insect-specific flaviviruses (ISFs); Phasi Charoen-like virus (PCLV); Aedes anphevirus (AeAv); Aedes albopictus densovirus (AalDNV-1); Aedes albopictus negev-like virus (AalNLV); Ross River virus (RRV); Barmah forest virus (BFV); LaCrosse virus (LACV); vesicular stomatitis virus (VSV); Culex pipiens densovirus (CpDV); Rift Valley fever virus (RVFV); Japanese ecephalitis virus (JEV); Drosophila C virus (DCV); Flock house virus (FHV); Cricket paralysis virus (CrPV); Bluetongue virus (BTV); Nora virus (NV); Insect iridescent virus 6 (IIV-6)....;

Host Genus	Host Species	Wolbachia strain(s)	Virus blocked	Virus enhanced	Virus unaffected
*Aedes*	*aegypti*	*w*Mel	DENV [4–27]; ZIKV [7,28–31]; CHIKV [31–33]; YFV [33,34]; WNV [6]; MAYV [35,36]; SFV [7]; CFAV [37]; KUNV [5,37]	Putative ISFs [38]	PCLV [37]
		wMelCS	DENV [13,39]		
		*w*MelPop	DENV [10,12,21,22,40,41]; CHIKV [40]; YFV [33]; WNV [42]; CFAV [43,44]	AeAv [45]; AalDNV-1 [46]	PCLV [37,43]
		*w*AlbB	DENV [7,13,47–50–52]; ZIKV [7]; SFV [7]; CFAV [53]; AalNLV [53]	AalDNV-1 [46]	
		*w*Mel*w*AlbB	DENV [12]		
		*w*Au	DENV [7]; ZIKV [7]; SFV [7]		
		*w*Ri	DENV [39]		
		*w*AlbA	ZIKV [54]		SFV [7]
		*w*Pip			DENV [5]; KUNV [5]
	*albopictus*	*w*AlbAwAlbB†	DENV [55,56]; CHIKV [57]		DENV [58]
		*w*AlbB†	DENV [51,59]; ZIKV [59,60]; CHIKV [61,62]; WNV [59]; RRV [59]; BFV [59]	AalDNV-1 [46]	
		*w*Mel	DENV [63,64]; CHIKV [62,64,65]		
		*w*MelPop	DENV [66,67]		
		*w*AlbA*w*AlbB*w*Au	DENV [68]; ZIKV [68]		
		wStri	DENV [69]; ZIKV [60,69]; CHIKV [69]; YFV [69]		LACV [69]; VSV [69]
		*w*AlbA*w*AlbB*w*Pip	DENV [70], ZIKV [70]		
		*w*Pip			DENV [5], CHIKV [5]
		*w*Pip*w*Mel	DENV [64], CHIKV [64]		ZIKV [64]
	*polynesiensis*	*w*AlbB	DENV [71]		
	*fluviatilis*	*w*Flu†			DENV [72]
	*notoscriptus*	*w*Noto†			DENV [73]
*Culex*	*quinquefasciatus*	*w*Pip†	WNV [74]	CpDV [75]	
	*tarsalis*	*w*AlbB‡		WNV [76]	RVFV [77]
*Armigeries*	*subalbatus*	*Unclassified*			JEV [78]
*Drosophila*	*melanogaster*	*w*Mel†	DENV [79]; DCV [80–85]; FHV [80–82,84,86]; WNV [74]; CrPV [81]; BTV [87]; NV [80]; SFV [88]; SBV [82,89,90]		IIV-6 [80]; KV [91], multiple [92,93]
		*w*MelCS†	DCV [82,94–97]; FHV [95]		
		*w*MelPop†	DENV [79,98]; DCV [82]		
	*simulans*	Multiple, see [99,100]	DCV [99,100]; FHV [100]		DCV [99,100]; FHV [100]
		*w*San		FHV [100]	
		*w*Ha		DCV [100]	
	*suzukii*	*w*Suz†	DCV [101]; FHV [101]		
	*pandora*	*w*PanCI†		CrPV [102]§	
*Cimex*	*lectularius*	*Unclassified*†			FCV [103]
*Nilaparvata*	*lugens*	*w*Stri	RRSV [104]		
*Spodoptera*	*exempta*	*w*Exe1-3†		SpexNPV [105]§	
*Varroa*	*destructor*	*Unclassified*†		DWV [106]	DWV [106]

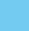
 (-)sense ssRNA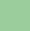 (+)sense dsDNA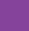 dsDNA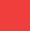 dsRNA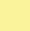 ssDNA

Key: †(native Wolbachia infection); ‡(transient non-germline Wolbachia infection); §(virus enhancement effect results from reduced host tolerance to infection rather than increased viral titers).

Note: Kallithea virus (KV); Feline calicivirus (FCV); Rice ragged stunt virus (RRSV); Spodoptera exempta nucleopolyhedrovirus (SpexNPV); Deformed wing virus (DWV).

Two distinct strategies are currently being deployed to utilize *Wolbachia* as a vector control intervention. Firstly, the spread of virus-blocking *Wolbachia* strains through mosquito populations can reduce their competence for certain arboviral diseases [47]. Secondly, wild populations can be suppressed by releasing males carrying *Wolbachia* strains that cause CI, and therefore sterility, when mated with wild females [70,112–114]. The former strategy, which is the focus of this review, aims for long-term replacement of wild populations with virus-blocking *Wolbachia*-carriers, while the latter attempts to achieve the local elimination of vector populations. Trials with artificial transinfections in the major arbovirus vector *Aedes aegypti*, which is not a natural *Wolbachia* host, have generated promising results with both approaches.

Releases in Northern Australia and Vietnam with *Ae. aegypti* carrying the *Wolbachia* strain *w*MelPop were unable to achieve stable and persistent population replacement, likely a result of the high fitness costs associated with this high-density strain [115]. *Ae. aegypti* carrying the lower density *w*Mel strain were released in Cairns, Australia, a city that experienced a relatively low number of locally acquired dengue cases annually. *w*Mel spread swiftly in the city and was maintained at proportions close to fixation, with case notification data indicating a reduction in dengue incidence of 96% [4]. Releases in 2014 of *w*Mel *Ae. aegypti* in the cities of Yogyakarta, Indonesia and Niteroi, Brazil met with similar success, with estimated average decreases in dengue incidence in release sites of 77% and 69%, respectively [116,117]. Releases of *w*Mel *Ae. aegypti* are also ongoing in sites in several additional countries including Columbia, Mexico, Fiji, and Sri Lanka. However, not all releases have resulted in the successful in establishment of *w*Mel; in Nha Trang City in central Vietnam the initial establishment of *w*Mel was followed by seasonal fluctuations in frequencies associated with elevated temperatures and the infection was subsequently lost from two release site areas [118]; furthermore, heatwave temperatures in the Australian city of Cairns were associated with reductions in *w*Mel frequencies [119].

*Wolbachia* strain *w*AlbB has been successfully deployed for dengue control in Greater Kuala Lumpur, Malaysia [47], releasing *w*AlbB-carrying *Ae. aegypti* in a variety of sites comprising different urban landscapes/building types (e.g. high-rise apartment buildings and landed houses) with intervention sites also selected for persistently high dengue incidence over preceeding years. Monitoring of the *Ae. aegypti* population size indicated no major release-related increases in population density, which is likely to be the result of compensatory CI-induced sterility between released *w*AlbB males and wild females. *w*AlbB invaded wild populations rapidly and reached a frequency of more than 90% in all intervention sites. After the cessation of releases, *w*AlbB frequencies remained stable in a majority of sites, while a minority experienced fluctuations and frequency drops that were largely overcome by additional low-level releases. The presence of *w*AlbB was associated with 40–85% decreases in dengue cases when comparing pre-intervention with post-inervention incidence over multiple years, although this is expected to be an underestimate of the true impact on transmission, given that dengue infection could be acquired by the routine travel of residents outside of the release areas [47].

A large body of experimental data has been published in recent years demonstrating the virus-inhibiting effect that *Wolbachia* can confer on host insects (see Table 1). This antiviral effect appears to be primarily active against viruses with a positive-sense single-stranded RNA genome [(+)RNA], which includes the mosquito-borne viruses most important to public health (dengue, Chikungunya, Zika, Yellow fever, West Nile etc.). Although an overwhelming majority of studies indicate that some combinations of host species and *Wolbachia* strain result in an inhibitory effect on viral replication (likely in part a reflection of a bias in the use of (+)RNA human arboviral pathogens in research studies), a small number of reports suggest that *Wolbachia* can in some circumstances enhance host susceptibility to infection with certain viruses. There is substantial divergence in insect host species and virus phylogeny examined in the various *Wolbachia*-virus interaction studies, as well as differences in experimental methodology, ranging from purely correlative studies to those with controlled laboratory infectious bloodmeal challenges. Among the laboratory studies, there is significant variation in the methods used to deliver virus, quantify virus, and even in the nature of the *Wolbachia* transinfection (e.g. stable germline or transient somatic infection). As any potential for viral enhancement is of clear public health importance for the use of *Wolbachia* in vector control, claims of enhancement should be closely examined and any implications carefully considered.

## Wolbachia-Mediated viral inhibition

*Wolbachia-*mediated protection from infection with pathogenic viruses, conferring increased survival, was first demonstrated in *Drosophila melanogaster* carrying the *w*Mel *Wolbachia* strain. *w*Mel-carrying flies displayed reduced mortality following challenge with (+)RNA viruses: *Drosophila* C virus (DCV), Nora Virus and Flock House virus [80], and which was associated with reductions in viral load, indicating that *Wolbachia* could inhibit viral replication [80,99]; *w*Mel and *w*Mel-Pop strains were also shown in to protect *Drosophila* against cricket paralysis virus [81]. It was found that this antiviral effect did not extend to Insect Iridescent Virus 6 (IIV-6), a virus with a double-stranded DNA genome – suggesting that the protective effect was restricted to viruses with certain genome replication modalities [80]. *Drosophila simulans* was subsequently used to test the protective capacity of an array of *Wolbachia* strains, demonstrating substantial differences in virus inhibition between *Wolbachia* strain variants [100].

Numerous *Wolbachia* transinfections were generated in *Ae. aegypti* and several showed a potent capacity to inhibit the transmission of dengue and other arboviruses that cause human disease (Table 1). Several factors are known to influence viral inhibition by *Wolbachia* in mosquitoes. To achieve transmission by a mosquito vector, virus present in a bloodmeal must first cross the cells of the midgut epithelium and establish an infection in the salivary glands. As evidence suggests that virus-blocking requires *Wolbachia*-virus coinfection in the same host cell [40,90] (although systemic factors activated by *Wolbachia* may also play a role in blocking in some hosts [120,121]), the presence of high *Wolbachia* densities in midgut and salivary gland tissues is likely to be critical for a strong virus-inhibition phenotype. *Wolbachia* strains vary widely in their somatic tissue distribution; in native infections, for example, *Wolbachia* tends to be primarily confined to the reproductive tissues, with viral inhibition correspondingly low or absent [58]. Non-native strains in contrast usually show wider tissue distribution with higher *Wolbachia* levels in somatic tissues [122,123]. However, there also appear to be fundamental differences between *Wolbachia* strains in their innate blocking capacity, which are independent of somatic density. The *w*AlbA and *w*Pip strains, for example, reach high levels in the salivary glands and midguts of *Ae. aegypti*, but have a modest impact on virus replication [5,54].

**Figure 1.**
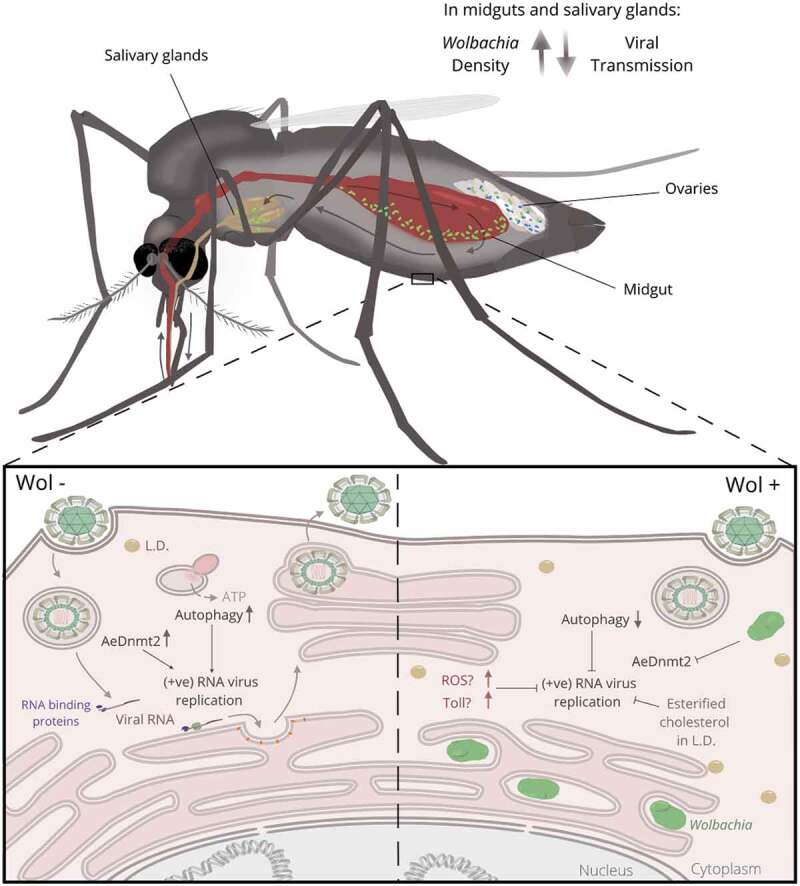
Distribution of the intracellular bacterium *Wolbachia* in mosquito hosts and some potential mechanisms that may be involved in the inhibition of arboviral transmission. Green spots in the mosquito salivary glands, midgut and ovaries represent the broad *Wolbachia* distribution typically seen in non-native *Wolbachia*-mosquito combinations. Blue spots restricted to the mosquito ovaries represent the limited *Wolbachia* distribution typically seen in native *Wolbachia*-mosquito combinations.

Although a complete mechanistic description of *Wolbachia*-mediated virus blocking remains elusive, there are strong indications of which processes are likely to be involved (Figure 1). An early hypothesis that a priming of host innate immunity was responsible was shown to be incomplete when it was observed that virus blocking was conserved in some native *Wolbachia-*host symbioses in the absence of innate immune activation [63,79,98,124,125]. Research has since focused on the various and sometimes extensive modifications of the host intracellular environment by *Wolbachia*, particularly the interactions between the host, *Wolbachia*, and virus, and the availability of certain classes of host lipid.

Within the host cell *Wolbachia* are located in cytoplasmic vacuoles comprising several layers of host-derived membrane [126]. Sequencing of several *Wolbachia* genomes indicates an inability for the symbiont to metabolize some membrane components, and therefore a reliance on the host for many of the materials needed for membrane generation [127]. Lipidomic analysis of *Wolbachia*-infected mosquito cells indicates a depletion of host sphingolipids and ceramides [128], while proteomic analysis suggests a disruption in vesicular trafficking that may impact the cellular capacity for sphingolipid processing [41]. *Ae. aegypti* cells infected with the *w*MelPop strain were also found to form lipid droplets enriched with esterified cholesterol, which was accompanied by reductions in free cellular cholesterol; experimental treatment with lipophilic cyclodextrins led to a dispersal of these lipid droplets and resulted in the partial rescue of dengue virus (DENV) replication without affecting *Wolbachia* density [41].

Other mechanisms that may contribute to viral inhibition include the production of reactive oxygen species (ROS) induced by the presence of *Wolbachia*, which has been linked to activation of the Toll innate immune pathway and the production of antimicrobial peptides in *Ae. aegypti* [129]. There is also evidence to suggest that *Wolbachia* may modulate host cell autophagy [130,131]. Increased autophagic turnover can reduce *Wolbachia* densities through the fusion of *Wolbachia*-containing endosomes with autophagic lysosomes [130]; it is therefore possible that autophagy may be suppressed by *Wolbachia*. DENV has been shown to induce autophagy in mammalian cells [132], which results in the processing of lipid droplets and the subsequent generation of ATP through β-oxidation, creating an energetic environment favorable for viral replication [132]. Hence, there may be antagonism between *Wolbachia* and virus over the regulation of cellular autophagic flux.

RNA-binding proteins (RBPs) play a critical role in the life cycle of RNA viruses and can have both pro and antiviral effects [133]. One such RBP is the DNA/RNA methyltransferase Dnmt2. In *Drosophila*, Dnmt2 actively binds DCV RNA and Dnmt2 mutant flies show elevated levels of DCV, suggesting an anti-viral role in this species [134]. *Drosophila* carrying the *w*Mel *Wolbachia* strain have increased Dnmt2 expression, and *w*Mel-carrying flies that are Dnmt2 mutants show reduced inhibition of Sindbis virus (SINV) [89]. In contrast however, *Wolbachia*-carrying *Ae. albopictus* and *Ae. aegypti* show reduced levels of AaDnmt2 [135,136]. Overexpression of AaDnmt2 in *Ae. aegypti* cells results in increased levels of DENV in *Wolbachia-*negative cells and AeDnmt2 has been shown to be pro-viral in both *Ae. aegypti* and *Ae. albopictus* for various Alphaviruses. Overexpression of AeDnmt2 in *A. albopictus* cells containing *w*Mel resulted in reduced viral inhibition of chikungunya virus (CHIKV) and SINV. Furthermore, viral RNA produced in cells containing *Wolbachia* show a significant reduction in m5C methylation [136], which may help explain why *Wolbachia*-carrying cells produce less infectious virus.

The RBP exoribonuclease 1 (×RN1) has also been implicated in *Wolbachia*-mediated antiviral activity [6]. Part of the RNA decay pathway, XRN1 actively degrades some flaviviral RNAs leading to the accumulation of sfRNAs, which in turn reduces XRN1 activity as degradation stalls; it remains bound to the subgenomic flavivirus RNA (sfRNA), effectively inhibiting the enzyme [6]. This leads to a favorable environment for viral RNA replication. When *Wolbachia* is present however, there is less viral replication and thus a reduced accumulation of XRN1-inhibiting sfRNAs and a more rapid degradation of viral RNAs by XRN1, likely enhancing the antiviral effects of *Wolbachia*.

A highly conserved feature of (+)RNA virus replication is a dependence on the formation of subcellular compartments through virus-induced rearrangements of organelle membranes, including those of the mitochondria, endosomes, or endoplasmic reticulum [137], which provide a scaffold for virus replication, physically containing the replication apparatus and shielding virus components from host immune factors. Several classes of lipid (particularly sphingolipids, and sterols including cholesterol) play key roles in determining membrane flexibility and rigidity [138,139]; ensuring appropriate membrane-lipid composition is critical to the promotion of membrane deformation and the assembly and function of (+)RNA virus replication complexes. There is evidence that some viruses even manipulate host lipid synthesis and transport pathways to promote the local enrichment of target lipids at sites of replication [140,141]. The inhibition of a range of (+)RNA viruses and evidence of an absence of inhibition of other viral genome types (see Table 1) that either replicate in the nucleus (DNA viruses) [80,91] or replicate in the cytoplasm but do not directly require the formation of membranous compartments for replication (double stranded and negative-sense single-stranded RNA viruses) [37,43,69], is therefore consistent with the hypothesis that virus blocking is primarily due to a perturbation by *Wolbachia* of pathways involved in the synthesis or localization of membrane lipids. Although not currently fully understood, there are likely to be fundamental mechanistic reasons for disparities in inhibition between different virus types, with evidence suggesting that those viruses most affected will be those most sensitive to *Wolbachia-*induced perturbations in lipid homeostasis.

## Prospects for long-term efficacy of Wolbachia transmission-blocking

There are some important implications of the research published to date on the mechanistic basis of *Wolbachia*-mediated virus inhibition for the long-term prospects for the use of *Wolbachia* in dengue control. Firstly, the likelihood that multiple cellular perturbations contribute to virus inhibition, while making the phenotype more challenging to experimentally dissect, does reduce the possibility that viral escape mutations will arise that restore DENV replication. Indeed, no examples of DENV escape mutants have to date been reported. This apparently built-in robustness is analogous to multidrug therapy using drugs with different modes of action to reduce the likelihood of evolution of pathogen drug resistance. Nevertheless, differences between *Wolbachia* strains should also be considered in this context. The *w*Mel strain in *Ae. aegypti* was found to be unstable when larvae were reared at elevated temperatures (diurnal temperatures with maxima above 34°C) [7,142], with large decreases in density in host tissues, leading to reduced maternal transmission and penetrance of cytoplasmic incompatibility [143], and substantially reducing its ability to inhibit DENV transmission [144]. In contrast, *w*AlbB was stable at the high rearing temperatures used. This affects the relative utility of different strains of *Wolbachia* for dengue control in very hot climates – an important factor for long-term robustness of the approach in the face of climate change. Furthermore, the use of strains with high-temperature stability could also ensure that any risks of the selection of virus escape mutations are minimized. This is analogous to the concept that drug dose and treatment duration affect the risk of selection of drug resistance, with a ‘mutant prevention concentration’. More research is needed on the effects of high temperatures on the *Wolbachia*-induced cellular perturbations relevant to virus replication. It is important to note that no virus escape mutations have been reported to date.

A second area where greater knowledge of the mechanisms of *Wolbachia-*mediated virus inhibition will be very useful is for assessing the implications of any *Ae. aegypti-Wolbachia* co-evolutionary changes that could occur over time to minimize host fitness costs. For example, virus transmission blocking could be reduced over time if mosquito-*Wolbachia* co-evolution results in lower *Wolbachia* density overall, or more restricted tissue distribution to the ovaries and testes. The most important *Wolbachia*-associated costs detected in *Ae. aegypti* are in reduced hatch of embryos following quiescence (dry storage) [145], and reduced fertility of females that result from quiesced eggs [146]. Thus, selection on these traits could act specifically on the ovaries and embryos and may not have any effects in tissues relevant to DENV replication and transmission, namely the midgut and salivary glands. Again, further research is needed on the mechanisms of fitness reduction in *Ae. aegypti* and its relationship to virus inhibition. It is also important to note that no evidence for density reduction or loss of virus transmission-blocking capacity has been reported to date in *Ae. aegypti* field populations tested several years after *Wolbachia* introduction [8,48,147].

In addition, there is mounting evidence that introgressing *Wolbachia* strains into *Ae. aegypti* genetic backgrounds from different geographical areas does not have a major impact on *Wolbachia* density [148] or the capacity to block virus transmission – indicating a robustness of the phenotype with varying host genotype [149,150]. Artificial selection experiments aimed at generating host genotype lineages displaying either high or low levels of virus blocking linked weaker blocking to reductions in overall host fitness, suggesting that selection in the field may act to maintain high levels of virus blocking strength [9].

## Evidence for Wolbachia-mediated viral enhancement

### Enhancement of viruses with positive-sense single-stranded RNA genomes

The first published study describing *Wolbachia*-mediated enhancement of a (+)RNA virus is from Dodson et al [76], and describes an increased infection rate of West Nile virus (WNV) *Culex tarsalis* mosquitoes transiently infected with the *w*AlbB *Wolbachia* strain. Following challenge with WNV, *Cx. tarsalis* transiently infected with *w*AlbB showed a significantly higher WNV infection rate compared to non-*Wolbachia* controls at 7-days post infection – although there was no effect of the *Wolbachia* infection on rates of viral dissemination to mosquito legs or transmission in salivary secretions at this time point. A second time point (14-days post infection) found no significant differences in rates of infection, dissemination or transmission between *Wolbachia-*positive and *Wolbachia-*free controls.

As noted by the authors [76], a key caveat in the study is the transient nature of the *Wolbachia* infection. Although technically easier to generate, transient infections resulting from adult intrathoracic injection do not reliably recreate the *Wolbachia* densities or tissue distributions observed in natural germline transinfections [49]. Virus inhibition by *Wolbachia* appears to be largely cell autonomous, requiring the co-localization of *Wolbachia* and virus in the same host cell – although there may also be a systemic contribution in some cases in the form of immune activation [40,74,129]. Hence, the presence of high densities of *Wolbachia* in certain host tissues involved in viral infection and transmission, such as the midgut and salivary glands, is important for transmission blocking. While stable germ-line transinfections tend to produce stable midgut and salivary gland densities, transient infections are less consistent. Quantitative PCR analysis of *Wolbachia* titers in the Dodson study [76] suggested that *w*AlbB levels varied over 400-fold between individual transiently infected *Cx. tarsalis* mosquitoes (ranging from 400 *Wolbachia* per host cell to less than 1 *Wolbachia* cell per host cell, with most individuals displaying lower densities in the range of 0–1 *Wolbachia* cells per host cell), a level of variability far greater than that typically observed in germline infections [7]. Moreover, there did not appear to be a correlation between *Wolbachia* titer and probability of infection with WNV, as may be expected if *Wolbachia* was causing enhanced host susceptibility to virus infection. A separate study comparing WNV blocking in transient and germline *w*AlbB-infected *Ae. aegypti* found that transient somatic transinfection significantly underestimated levels of WNV inhibition compared to germline transinfection, although significant WNV inhibition was observed in both cases [49]. Several other studies have shown inhibition of WNV by *Wolbachia*, including the *w*AlbB strain in *Ae. albopictus* cells [59] and *w*Mel in *Ae. aegypti* cells [6], indicating that WNV is similar to other flaviviruses in its susceptibility to *Wolbachia*-mediated antiviral activity. While it is possible that *Cx. tarsalis* differs from other host species in its interactions with *Wolbachia* such that it causes viral enhancement instead of inhibition, the use of transient *Wolbachia* infections in the Dodson study [76] makes the drawing of definitive conclusions difficult. Moreover, while the *Wolbachia-*infected cohort was injected with a *w*AlbB-containing *An. gambiae* cell-line extract, the *Wolbachia*-negative cohort was injected with clean media – rather than the preferred control of a *Wolbachia*-negative *An. gambiae* cell-line extract. It is likely that a complex mixture of *An. gambiae* cellular debris/mitochondrial/insect specific virus (ISV) material was co-introduced into *Cu. tarsalis* along with *w*AlbB, with unknown effect on the host. The Dodson study [76] reports that the *Wolbachia*-injected cohort had a slight but significant downregulation of Rel1, a Toll pathway transcription factor important in the Toll-mediated innate immune response. This is surprising given that *Wolbachia*-derived cellular components such as the *Wolbachia* surface protein (WSP) can act as pathogen-associated molecular patterns (PAMPs) [151], and have been shown to activate the Toll immune pathway in species that do not carry native *Wolbachia* infections [121,129], such as *Cx. tarsalis*.

A further study reporting the enhancement of (+)RNA viruses by *Wolbachia* in a mosquito host concerns an increased infection rate of insect-specific flaviviruses (ISFs) in field-caught *Ae. aegypti* mosquitoes carrying the *w*Mel *Wolbachia* strain [38]. In this study, wild *Ae. aegypti* were sampled from sites in Cairns, Australia: *w*Mel-carrying mosquitoes were collected from two sites, while *Wolbachia*-free mosquitoes were sampled from a third site. Using specific primers, ISF fragments were PCR amplified and then sequenced. The authors reported a higher proportion of *w*Mel-carrying mosquitoes positive for known ISF sequences compared with *Wolbachia*-free mosquitoes. While this result is consistent with an ISF enhancement effect by *w*Mel, the limited number of sampling sites used, and the absence of both *Wolbachia* positive and negative samples from the same sites, leaves the possibility that differences may simply reflect geographical variation in ISF abundance and/or host background [152]. The authors also assessed the infection rate of known ISF sequences in laboratory mosquitoes reared under standard conditions but did not observe any differences between *w*Mel-carrying and *Wolbachia*-free mosquitoes. An analysis of ISF levels in mosquitoes of field and laboratory origin by qRT-PCR suggested a tendency for a stronger ISF signal in *w*Mel carriers, although there were no instances where significant differences in putative ISFs were consistent across both field and laboratory sampled mosquitoes, and in one of the five putative ISFs, both field and laboratory mosquitoes showed significant differences between *w*Mel-carriers and non-carriers, but with conflicting outcomes (i.e. titers of the putative ISF were significantly lower in *w*Mel-carriers than non-carriers in field-caught mosquitoes, but the reverse was true for those that were laboratory-reared). Unexpectedly, one of the sequences that was significantly more abundant in laboratory-reared *w*Mel-carriers showed high (99%) similarity to cell fusing agent virus (CFAV), an ISF for which strong inhition by *Wolbachia* has previously been shown [37,43,44].

Two studies report an enhancement effect of *Wolbachia* on viral infection in *Drosophila* species. Martinez et al. 2014 [100] tested germline transinfections with 19 *Wolbachia* strains in *Drosophila simulans*, and challenged the lines with *Drosophila* C virus (DCV) and Flock House virus (FHV), (+)RNA viruses from the Picornaviridae and Nodaviridae families, respectively. For DCV, 7 out of the 19 *Wolbachia* lines showed a significant reduction in viral titer compared to *Wolbachia*-negative controls, while one showed a significant increase. For FHV 5 out of the 19 lines showed a significant reduction in viral titer, while one showed a significant increase. Interestingly, the *Wolbachia* strain associated with increased DCV titer was not the same as the strain associated with increased FHV titer – each strain correlated with increased titers of one virus showed no significant interaction with the other.

Asselin et al. 2019 [102] characterized two native *Wolbachia* infections in *Drosophila pandora*: *w*PanMK and *w*PanCI. Challenge of flies with cricket paralysis virus (CrPV), a (+)RNA virus from the Family Dicistroviridae, revealed that *w*PanMK was associated with reduced CrPV-induced mortality, while *w*PanCI was associated with increased mortality compared to *Wolbachia*-negative controls. Measurements of viral titers showed no significant differences in CrPV levels between the fly lines (although only one timepoint was used), suggesting that the *Wolbachia* infections did not have a direct impact on CrPV replication, but rather appeared to affect the ability of *D. pandora* to tolerate the infection.

Grau et al. [106] investigated a potential correlation between the presence of a native *Wolbachia* infection and the frequency of deformed wing virus (DWV) in the parasitic honeybee mite *Varroa destructor*, with specimens collected from several different apiaries. Two sets of primers were used for *Wolbachia* detection in these samples, with the authors reporting a significant positive correlation between infection frequency with *Wolbachia* and DWV with one primer set but not with the other. As there was little agreement in infection frequency between the two primer sets (in one of the hives the different PCR assays produced a disparity in *Wolbachia* infection frequency in mites that ranged from 0% to 100%), there are concerns over the sensitivity and/or specificity of the PCR assays used in the detection of *Wolbachia*. It is possible that multiple strains of *Wolbachia* naturally infect *V. destructor*, which could explain the inconsistency between primers sets; however, it is difficult to draw definitive conclusions about potential correlations with DWV without more robust characterization of the *Wolbachia* present.

### Enhancement of viruses with DNA or negative-sense RNA genomes

In one of the first studies examining *Wolbachia-*virus interactions, Teixeira et al [80]. reported that the virus inhibition phenotype was not observed when *w*Mel-carrying *D. melanogaster* were challenged with Insect Iridescent Virus 6 (IIV-6), a large double-stranded DNA virus. A small number of studies have since investigated the effect of *Wolbachia* on DNA viruses. In one study [46], qPCR was used to positively correlate *Wolbachia* density with titers of the ssDNA *Ae. albopictus* densovirus (AalDNV-1) in cultured *Ae. aegypti* and *Ae. albopictus*-derived cell lines carrying the *w*MelPop or *w*AlbB *Wolbachia* strains. The authors found that under normal cell culturing conditions the *w*MelPop and *w*AlbB-infected cells contained approximately 5-20-fold more AalDNV-1 genomes per host cell than tetracycline-cured controls. Using cells cultured in media containing varying concentrations of tetracycline, the authors generated a range of *w*MelPop and *w*AlbB intracellular densities and found that *Wolbachia* levels positively correlated with AalDNV-1 genome copies. The authors hypothesized that elevated DNA repair response pathways induced by disruption of cellular redox homeostasis triggered by *Wolbachia-*generated reactive oxygen species may provide the additional molecular apparatus required for increased AalDNV-1 replication. Similarly, studies investigating the interaction of *Wolbachia* on the load of the ssDNA Culex pipiens densovirus (CpDV) on field caught samples found a positive correlation between CpDV levels and the density of the native *Culex pipiens Wolbachia* strain *w*Pip [75], and laboratory studies found that the vertical transmission rate of CpDV was higher in *Wolbachia*-positive mosquitoes than in antibiotic-cured controls [153]. A study on a newly characterized negative-sense RNA ISV named Aedes anphevirus (AeAV) investigated the effect of *w*MelPop-CLA on viral load in the *Ae. aegypti* Aag2 cell line [45]. A quantitative analysis of genomic viral RNA suggested a significant enhancing effect of *Wolbachia* compared to a *Wolbachia*-cured Aag2 cell line.

Three *Wolbachia* strains (*w*Exe1–3) native to the African army worm, *Spodoptera exempta*, were assessed for their effect on host infectivity with Spodoptera exempta nucleopolyhedrovirus (SpexNPV), a double-stranded DNA virus (family: baculovirus) that displays very strong host pathology [105]. The authors carried-out field sampling of *S. exempta* which suggested a positive correlation between the three *Wolbachia* infections and rates of SpexNPV-induced mortality. Follow-up laboratory-based bioassays using varying doses of SpexNPV on *w*Exe1-carrying and tetracycline-cured *S. exempta* larvae suggested that the *Wolbachia*-free group had a LD_50_ 6-14-fold greater than that of *w*Exe1 carriers. Interestingly however, while both the field and laboratory findings indicated increased larval mortality associated with *Wolbachia* infection, quantification of SpexNPV in dead larvae revealed a tendency for lower numbers of viral occlusion bodies in the *Wolbachia* carriers. This may be due to a faster ‘speed of kill’ of the virus in *Wolbachia*-carriers, resulting in less time for occluded viral forms to accumulate in moribund larvae, and may result from a lower tolerance of *Wolbachia-*carriers for viral infection.

## Implications of viral enhancement on the use of Wolbachia in vector control

*Wolbachia* are being deployed in both population suppression and population replacement vector control strategies. Population replacement strategies aim to spread and maintain an introduced *Wolbachia* strain in a wild population; any virus enhancement is therefore of potential interest to public health. Currently, the spread of novel *Wolbachia* strains through wild populations has been limited to *Ae. aegypti -* although promising strains have also been developed for *Ae. albopictus* [65,68]. The overwhelming majority of studies assessing *Wolbachia*-virus interactions have investigated novel transinfections in *Ae. aegypti* in both *in vivo* and cell culture systems and have used a wide variety of arboviruses, focusing on the major human pathogenic viruses belonging to the virus families *Flaviviridae* (genus *Flavivirus*) and *Togaviridae* (genus *Alphavirus*). These arboviruses contain positive-sense single-stranded RNA genomes and are responsible for the vast majority of arboviral morbidity and mortality globally [154]. Studies have shown that the effect *Wolbachia* can have on viral replication varies depending on multiple factors including the *Wolbachia* strain, the host species, and the replication modality of the virus. However, only a single study out of more than 50 involving *Ae. aegypti* and *Ae. albopictus* reports evidence of enhancement of viruses known to cause pathology in humans. This study, by King et al., 2018 [155], reexamines data from two previous publications that use a variety of dengue titers in challenges of *w*Mel-carrying *Ae. aegypti* [10,156], and use statistical modeling to conclude that *w*Mel can increase susceptibility to mosquito dengue infection when challenge titers are low. However, the data used to generate one of the primary data sets suffers from various shortcomings, including low statistical power and the potential for false positives when challenges were performed at low viral titers, and issues with the statistical model used. For a more thorough examination of the King et al., 2018 [155] study see Ant et al., 2020 [157].

Although only a single study in *Aedes* mosquitoes reports the enhancement of a pathogenic human virus, several report elevated ISV titers. ISVs do not replicate in vertebrate cells, and therefore do not pose a direct public health concern themselves, although they do have the potential to interact with and modulate the replication of human arboviruses when co-infecting the same mosquito host, primarily through mechanisms of superinfection exclusion – a process where a host cell infected with a virus has a reduced capacity to support the productive replication of a secondary viral infection. Superinfection exclusion has been observed between several ISVs and human arboviruses, including with WNV and the insect-specific Culex flavivirus (CxFV), where experimentally challenged *Culex pipiens* mosquitoes showed reduced WNV dissemination early in infection when superinfected with CxFV [158] – although a separate study failed to find an effect of CxFV on WNV replication in *Culex quinquefasciatus* [159]. *Ae. aegypti* cells infected with *Wolbachia* were associated with higher titers of Aedes anphevirus (AeAV), an ISV that showed a mild suppressive effect on DENV [45,60] and ZIKV [160] replication in co-infected cells *in vitro*. Interestingly, superinfection exclusion does not appear to be limited to viruses with similar genomes/replication modalities. As discussed above, *Wolbachia* presence was correlated with enhanced replication of the ssDNA virus, Aedes albopictus densovirus (AalDNV-1), in *Ae. aegypti* cells. A previous study of an Aedes albopictus densovirus (AalDNV) found that AalDNV could restrict the replication of DENV in *Ae. albopictus* cells, despite AalDNV localizing its replication to the nucleus and DENV to the cytoplasm [161]. Assuming similar enhancement of persistent ISVs by *Wolbachia* in field populations of *Aedes* mosquitoes, current evidence suggests that mechanisms of superinfection exclusion may actually contribute to reduced transmission competency of *Wolbachia*-carriers.

Some studies suggest that *Wolbachia* may reduce the tolerance of host insects to pathogenic ISVs [102,105]. A reduction in host tolerance to a given pathogenic ISV could reduce the relative fitness of *Wolbachia*-carriers and therefore influence the invasiveness or population stability of a strain introduced for arbovirus control. However, there are also numerous examples of host protection against pathogenic ISVs. Protection from pathogenic ISVs appears to be the more common phenotype and has been suggested as a mechanism by which *Wolbachia* strains can spread from very low initial infection frequencies following natural horizontal transmission events, when the fitness advantages from frequency-dependent CI are negligible [152].

## Conclusions

*Wolbachia* can modulate the susceptibility of host cells to infection with some viruses, with the magnitude of this effect varying widely between *Wolbachia* strains and host species. While the altered intracellular state may be more favorable to the replication of some virus types, current evidence overwhelmingly indicates that *Wolbachia* has an inhibitory effect on the replication of (+)RNA viruses – which includes the vast majority of human arboviral pathogens. Although there are some rare examples of *Wolbachia* enhancing (+)RNA virus replication in insects, these are very much in the minority, and there are often significant caveats to the experimental design or interpretation of results in these studies. The effects of *Wolbachia* on viruses without (+)RNA genomes appear to be more nuanced. The vast majority of these involve ISVs, the modulation of which may influence arboviral disease transmission through mechanisms of superinfection exclusion or by altering the fitness/pathogen tolerance of mosquito hosts.
